# The Politics of Disease Epidemics: a Comparative Analysis of the SARS, Zika, and Ebola Outbreaks

**DOI:** 10.1007/s40609-018-0123-y

**Published:** 2018-09-03

**Authors:** Lydia Kapiriri, Alison Ross

**Affiliations:** Department of Health, Aging and Society, McMaster University, 1280 Main Street West, Hamilton, ON L8S 4L8 Canada

**Keywords:** Politics, Epidemics, SARs, Zika, Ebola

## Abstract

Over the past few decades, disease outbreaks have become increasingly frequent and widespread. The epicenters of these outbreaks have differed, and could be linked to different economic contexts. Arguably, the responses to these outbreaks have been “political” and inherently burdensome to marginalized populations. Key lessons can be learned from exploring the narratives about the different epidemics in varying income settings. Based on a review of the published medical, social, and political literature, which was accessed using four electronic databases—PubMed, Sociological Abstracts, Scholars Portal, and Web of Science, the overall objective of this paper discuss scholars’ narratives on the “politics” of Ebola in a low-income setting, Zika virus in a middle-income setting, and SARS in a high-income setting. Various themes of the politics of epidemics were prominent in the literature. The narratives demonstrated the influence of power in whose narratives and what narratives are presented in the literature. While marginalized populations were reported to have borne the brunt of all disease outbreaks in the different contexts, the prevalence of their narratives within the reviewed literature was limited. Regardless of income setting, there is a need to give voice to the most marginalized communities during an epidemic. The experiences and narratives of those most vulnerable to an epidemic—specifically poor communities—need to be represented in the literature. This could contribute to mitigating some of the negative impact of the politics in epidemics.

## Introduction

Infectious diseases are responsible for 25% of the annual global deaths (Dry and Leach 2010). Epidemics arising from these diseases are unpredictable, carry uncertain, varying risks, and narratives in different contexts (Scoones 2010). It is important that the scholarly literature represents the diverse and sometimes competing, narratives from all affected particularly the most vulnerable (Dry and Leach 2010). Arguably, national and global responses to epidemics are inherently political. The experts selected for consultation, the evidence used to inform response pathways, and narratives of blame, vulnerability, and responsibility are politically driven, and require analysis.

In this paper, *epidemics* refer to a spike, above normal, in the prevalence of a specific disease in a specific population (Centre for Disease Control and Prevention 2012). The literal definition of *politics* is the “activities that relate to influencing the actions and policies of a government or getting and keeping power in a government” (Merriam-Webster 2016). However, in the “politics of epidemics” literature, politics often refers to the extensive and diverse influence of local, national, and international governments and organizations, on the health outcomes of communities during disease outbreaks. For the purposes of this paper, politics will also refer to how scholars talked about epidemics, and whose narratives are reported in the reviewed literature.

Several key themes emerge when examining scholarly narratives about the politics of epidemics namely the socioeconomic distribution of disease, decision-making in research and development, the credibility of evidence that informs response pathways, and attribution of responsibility for causing the outbreak and determining who is responsible for responding. While the literature presents obviously competing narratives that explain disease outbreaks, a close examination of these reveals a high prevalence of certain narratives, which reflect the influence of power and privilege. Hence, some of the literature has called for increased representation of the narratives from the most marginalized populations who bear the brunt of epidemics, in the scholarly literature (Dry and Leach 2010; Nightingale 2010).

Furthermore, the decisions with regard to the type of research, the study population and the research beneficiaries often lack transparency, are also dominated by the most powerful. The case of the swine flu, dubbed the “pandemic that never really was” (Evans 2010; Bonneux and Van Damme 2010), demonstrates the potential consequences of private and uncommunicated decision-making processes in research and development. In the case of the swine flu, allegedly scientists conducting research and advising the World Health Organization on the virus were compensated by drug companies. This may have introduced bias (Evans, 2010, p. 296). By increasing fear of a global pandemic, scientific research funded by pharmaceutical companies justified the stockpiling of anti-viral supplies, thus increasing their vaccine sales (Evans 2010).

Fear mongering has been criticized in the literature, since it has the potential to distort the evidence, which should guide credible decision-making. It is therefore critical to assess the credibility and quality of the evidence that is being used to inform the response to epidemics around the world, particularly in light of the complicated and often problematic relationship between the academic and industry (Bonneux and Van Damme 2010). In determining the credibility of evidence, consideration should be given to the legitimacy and authority of its producer(s).

The literature identifies discourse with regard to the attribution of responsibility; who is to blame for the outbreak and who should response? Some of this literature assigned the blame and responsibility, for the outbreak, to people’s cultures (Dong 2008; Morinet et al. 2016), which oversimplifies complex issues that result from complex processes, such as globalization, climate change, and global economics. This kind of narrative reflects the politics of blame, which typically attributes responsibility for the sources of the outbreak to a cultural minority group and neglects the influence of colonialism and the social determinants of health, including overcrowding, poor sanitation, and poverty that increase these people’s vulnerability (Dry and Leach 2010).

Given these sociopolitical issues that characterize epidemics, a comparative analysis of the narratives on the politics of epidemics is relevant. To date, most of the narratives in the scholarly literature on the politics of epidemics have considered one epidemic in one context (Dong 2008; Morinet et al. 2016; Nyenswah et al. 2014). We hypothesize that the narratives may vary depending on the kind and context of the epidemic.

The overall aim of this review paper is to discuss the scholarly narratives on the politics of disease epidemics by diseases and income level; Ebola in a low-income setting, Zika in a middle-income setting, and SARS in a high-income setting (Table 1). This analysis allowed for the consideration of the role that socioeconomic, geographical, and cultural context might play in the narratives on the attribution of blame and response to disease outbreaks.

**Table 1:** An overview of the disease outbreaks

Outbreak	Income level of area of investigation	Year	Global magnitude	Case-specific magnitude
Ebola (World Health Organization, 2017e)	Low income—Liberia	2014	28,646 cases	10,675 cases
			11,323 deaths	4809 deaths
Zika (Summers, 2009)	Middle income—Brazil	2015–ongoing	83,760 cases	66,180 cases
			9 deaths *(PAHO, 2016)*	4 deaths
SARS	High income—Toronto, Canada	2003	8096 cases	251 cases
			774 deaths	43 deaths

## Methods

The paper is based on a review of the *peer-r*eviewed published medical, social, and political literature, which was accessed using four electronic databases—PubMed, Sociological Abstracts, Scholars Portal, and Web of Science. The search was limited to *full text articles* published between 2002 and 2017.

A similar approach was used in identifying the relevant articles. For each epidemic, the search terms included the name of the disease outbreak and the country of interest: “SARS AND Toronto,” “Zika AND Brazil,” and “Ebola AND Liberia.” It was vital that each search included the income setting to ensure that the findings were contextually relevant. These terms were then combined with the terms “societ*,” “sociol*,” and “politic*.” Truncation was used to ensure inclusion of all terms, including “society,” “societal,” “sociological,” “sociology,” “politics,” “political,” “politician,” and “politicization.” The search terms must have appeared in the title or abstract, except the terms “sociol*,” “societ*,”,and “politic*” which could appear “anywhere”. The Web of Science database allowed for more customized filtration; therefore, “document type” was set to article, “research domain” was set to “social sciences,” and research areas were refined to “sociology,” “government,” “law,” “social sciences” other topics, “biomedical social sciences,” “social issues,” and “public administration.” The Scholars Portal database allowed for similar filtration by subject, which was set to social sciences.

Tables 2, 3, and 4 summarize the search results for the three outbreaks within the specified contexts. The titles and abstracts of the search results were reviewed for relevance to the politics of epidemics. All biomedical articles describing biomedical research were excluded. All articles that were relevant to the study topic were retrieved and reviewed. The initial review involved RA, grouping the search results according to the disease outbreak. For each outbreak, RA first scanned through a couple of papers, identifying the emerging themes. Once these were identified, subsequent reviews were structured along these themes, although an open stance was maintained throughout to enable the reviewer to identify any additional relevant themes.

**Table 2:** Search words and number of hits for SARS

Database	Search terms	Results	Relevant results
PubMed	“SARS” AND “Toronto”	156	11
	“SARS” AND “Toronto” AND “sociol*”	0	0
	“SARS” AND “Toronto” AND “societ*”	4	0
	“SARS” AND “Toronto” AND “politic*”	1	1
Sociological abstracts	“SARS” AND “Toronto”	9	4
	“SARS” AND “Toronto” AND “sociol*”	5	4
	“SARS” AND “Toronto” AND “societ*”	2	1
	“SARS” AND “Toronto” AND “politic*”	5	1
Scholars portal	“SARS” AND “Toronto”	11	5
	“SARS” AND “Toronto” AND “sociol*”	N/A	N/A
	“SARS” AND “Toronto” AND “societ*”	N/A	N/A
	“SARS” AND “Toronto” AND “politic*”	N/A	N/A
Web of science	“SARS” AND “Toronto”	52	12
	“SARS” AND “Toronto” AND “sociol*”	0	0
	“SARS” AND “Toronto” AND “societ*”	2	0
	“SARS” AND “Toronto” AND “politic*”	2	2
Total accessed/reviewed		249	41 (due to overlap in search results across databases)

**Table 3:** Search words and number of hits for Zika

Database	Search terms	Results	Relevant results
PubMed	“Zika” AND “Brazil”	378	17
	“Zika” AND “Brazil” AND “societ*”	8	2
	“Zika” AND “Brazil” AND “sociol*”	0	0
	“Zika” AND “Brazil” AND “politic*”	5	3
Sociological abstracts	“Zika” AND “Brazil”	1	0
	“Zika” AND “Brazil” AND “societ*”	0	0
	“Zika” AND “Brazil” AND “sociol*”	1	0
	“Zika” AND “Brazil” AND “politic*”	0	0
Scholars portal	“Zika” AND “Brazil”	1	0
	“Zika” AND “Brazil” AND “societ*”	N/A	N/A
	“Zika” AND “Brazil” AND “sociol*”	N/A	N/A
	“Zika” AND “Brazil” AND “politic*”	N/A	N/A
Web of science	“Zika” AND “Brazil”	10	1
	“Zika” AND “Brazil” AND “societ*”	0	0
	“Zika” AND “Brazil” AND “sociol*”	0	0
	“Zika” AND “Brazil” AND “politic*”	0	0
Total		404	23 (due to overlap in search results across databases)

**Table 4:** Search words and number of hits for Ebola

Database	Search terms	Results	Relevant results
PubMed	“Ebola” AND “Liberia”	367	37
	“Ebola” AND “Liberia” AND “societ*”	12	4
	“Ebola” AND Liberia” AND “sociol*”	2	2
	“Ebola” AND Liberia” AND “politic*”	9	3
Sociological abstracts	“Ebola” AND “Liberia”	11	4
	“Ebola” AND “Liberia” AND “societ*”	2	1
	“Ebola” AND Liberia” AND “sociol*”	4	1
	“Ebola” AND Liberia” AND “politic*”	8	3
Scholars portal	“Ebola” AND “Liberia”	9	4
	“Ebola” AND “Liberia” AND “societ*”	N/A	N/A
	“Ebola” AND Liberia” AND “sociol*”	N/A	N/A
	“Ebola” AND Liberia” AND “politic*”	N/A	N/A
Web of science	“Ebola” AND “Liberia”	2	1
	“Ebola” AND “Liberia” AND “societ*”	0	0
	“Ebola” AND Liberia” AND “sociol*”	0	0
	“Ebola” AND Liberia” AND “politic*”	2	1
Total		428	61 (due to overlap in search results across databases)

The four themes socioeconomic distribution of disease, decision-making in research and development, the credibility of evidence that informs response pathways, and the attribution of infectious disease responsibility, (outlined in the introduction), were used to guide the synthesis of the information from the literature. For consistency, detailed discussions are limited to the findings that are directly related to the politics of epidemics, as defined in this paper.

## SARS in Toronto

This review focused on the SARs epidemic in Canada, specifically Toronto, Ontario; to represent an epidemic in a high income country. Table 2 summarizes the search results.

The literature on SARS spoke to two of the four themes of the politics of epidemics, namely the credibility of evidence informing response pathways and the attribution of infectious disease responsibility.

## Credibility of Evidence Informing Response Pathways

Evidence is deemed credible if it is scientifically rigorous, and the legitimacy and authority of its producer. The credibility of the evidence used to guide some of the response decisions to SARS was questionable. Specific examples include the use of quarantine (Holm 2009; Jacobs 2007; Tracy et al. 2009) and travel advisories (Paquin 2007) both locally and globally.

The use of quarantine as a control measure, although considered to be highly effective, is controversial. A telephone-based survey in the Greater Toronto Area aiming to ascertain public perceptions of the use of quarantine found that while quarantine was perceived to be a necessary and effective strategy, its ethical implementation should involve the collaboration of policy-makers, public health organizations, and the general population, and should be closely regulated to ensure appropriate use and protection of individual rights (Tracy et al. 2009). Despite these recommendations, Toronto quarantined significantly more people during the SARS outbreak compared to the other affected cities, including Hong Kong and Shanghai (Jacobs 2007). Given the reported psychological distress reported by those quarantined, Toronto might have considered other strategies, such as the use of face masks to better “distribute the burden of containment measures” (Jacob, 2007, p. 532). Critics note that the extensive quarantining in Toronto lacked proper policies and procedures to guide its implementation (Jacobs 2007). Others highlight a lack of public record detailing any consultation between public health officials and the Ontario Human Rights Commission (Jacobs 2007). There was little public scrutiny, which was suggested to be the result of effective conditioning of the public consciousness to believe that quarantining recommendations would be made fairly and legitimately by senior public health officials (Jacobs 2007). Ultimately, it was not quarantining that was problematic, but the lack of apparent or sufficient evidence to guide its implementation.

Beyond extensive quarantining, The World Health Organization issued travel-advisories as an additional control measure to contain further national and international spread of SARS from Toronto (Paquin 2007). This travel-advisory cost Toronto $1.1 billion and restricted the international right for freedom of movement (Paquin 2007). Paquin (2009) criticizes these travel-advisories for various reasons. For example, the advisories were not made by the WHO in consultation with Toronto authorities and led to an uneven global distribution of the burden of SARS (Paquin 2007). Furthermore, the travel advisories were based on old data resulting from delayed communication between the federal government of Canada and the WHO, as information had to first travel from municipal to provincial to federal health authorities (Paquin 2007). Once again, the evidence used to inform the response was outdated and therefore considered unjustified. As such, there were problems with both the lack of quality evidence and the travel advisory as a response.

Quarantining and travel advisories reflect the profound ethical and political implications inherent in responding to infectious disease outbreaks. The evidence on the efficacy of the two primary responses—quarantining and travel advisories—was inadequate to justify the extent of their implementation.

## Attribution of Infectious Disease Responsibility

In the case of SARS in Toronto, the literature reported on both forms of responsibility. While some of the literature attributed the responsibility to the Canadian health care system for being unprepared to manage SARS, the other literature tended to (or report on the) how the public attributed blame to the Asian-Canadian community for “bringing” the outbreak. The latter narratives ultimately led to the racialization of the epidemic.

The narratives on attribution of responsibility for managing the SARs outbreak is most prevalent in the literature, with the limited capacity of the Canadian Health Care System to prepare for and respond to an emerging infectious disease as a main narrative. Many scholars pointed to flaws in the funding and organization of Canada’s health care system—federally, provincially, and municipally—to explain the SARS outbreak in Toronto. Some argued that information was communicated inefficiently and was often incorrect (Silas et al. 2006). Personal protective equipment was in short supply (Summers 2009), and the use of occupational health and safety in Toronto’s hospital system was inefficient, leading to further spread (Silas et al. 2006). Toronto’s hospital system was also blamed for lacking admission policies and public laboratories (Wong et al. 2007): an inability to supply health services when demand suddenly increased and a lack of quality leadership (Arya et al. 2009). Toronto was also ill-prepared to address the unique vulnerability of the homeless to SARS (Leung et al. 2008).

Inadequate collaboration between the various levels of government in Canada was blamed for the apparent inefficiencies and inadequacies in the functioning of the health care system and the response to public health crises. This led to disorganized contact tracing, quarantining, and communication to the public (MacDougall 2007). Financial challenges within the Canadian Health Care System further enhanced Toronto’s vulnerability to the SARS epidemic, including a lack of resourcing towards public health infrastructure and acute care (Salehi and Ali 2006). Similar to MacDougall (2007), Salehi and Ali (2006) point to a lack of cooperation and collaboration between the three levels of government to explain this public health crisis—ownership of responsibility and duty to respond was deflected between each level and remained unclear (MacDougall 2007; Salehi and Ali 2006).

Another sub-theme that emerged from the literature was the impact of globalization on local preparedness, prevention strategies, but more importantly, on the attribution of blame for the spread of the outbreak. While there is emphasis, in the narrative on SARs, for municipal governments to consider the globalized nature of the outbreak; (Keil and Ali 2007), consistent with the politics of epidemics literature, which asserts that blame is typically placed on cultural and ethnic minority groups, significant blame was placed on the Asian-Canadian community.

According to the literature, SARS quickly became a profoundly racialized disease and inflamed racial tensions in the Greater Toronto Area ultimately leading to the social exclusion of a racial minority—the Asian-Canadian group (Jacobs 2007). Such avoidance and stigmatization is reported to have played out in several spaces, such as on public transit and other public spaces, and families advising children to avoid Chinese peers in school (Jacobs 2007). Some of the literature posits that this racialized stereotyping could have been prevented with denunciation from leaders in government and public health (Jacobs 2007).On the other hand, according to Ali (2008), individualized health behaviors aimed at preventing SARS contraction—for instance, wearing a face mask—may have justified the avoidance of the stigmatized of the Asian-Canadians (Ali 2008).

Culture and ethnicity functioned not only as a risk factor for discrimination but also as a facilitator in the response to the outbreak. The Chinese-Canadian community in Toronto employed numerous strategies to combat SARS and ease social anxieties, including fundraising for research, the dissemination of health promotion materials, and launching a SARS support line, among other activities (Dong 2008). The mobilization of spiritual leaders was also found to be an effective means of disseminating public health information (Faust et al. 2009). While it is important to recognize the contributions of cultural and ethnic groups, we assert that cultural and ethnic minority groups are more often targets of blame, as was the case for SARS in Toronto.

## Zika in Brazil

Table 3 summarizes the total number of hits, and the number of papers that were retrieved and reviewed. The reviewed literature spoke to all of the themes identified in the politics of epidemics literature (socioeconomic distribution of disease, credibility of evidence, and the attribution of infectious disease responsibility), with the exception of decision-making in research and development.

## The Socioeconomic Distribution of Disease

Similar to the above outbreaks, the people who were most affected by Zika were in some way socially marginalized, the poor, and more specifically, poor women. At the global level, it was the poorer countries and communities within those regions that were most impacted: those with precarious and/or inconsistent access to health care services, lacking the resources and infrastructure to prevent, diagnose, and treat the virus (Plourde and Bloch 2016; Slavov et al. 2016). No wonder the impact of the outbreak was more devastating in Brazil, which was already financially strained prior to the emergence of the Zika virus, with limited human resources: doctors, nurses, and other specialists (Hennigan 2016), as compared to higher income countries who were more protected from the effects of the Zika virus given effective prevention programs, funding, and infrastructure (Gyawali et al. 2016). Easy and extensive access to mosquito repellants, air conditioning, effective waste management programs, and low rates of urban crowding protect more economically developed countries, such as the USA from Zika transmission (Plourde and Bloch 2016). Consequently, Zika virus has aptly been labeled an “infectious disease of poverty” (Gyawali et al., 2016, p. 3) (Gyawali et al. 2016).

Some of the literature attributed the lack of public health infrastructure and resources to respond to the Zika outbreak in Brazil to these inequalities with regard to who is most affected. Contrasting Zika with HIV, since HIV/AIDS initially affected the prestigious population, such as celebrities, doctors, scientists (Lotufo 2016), they were able to advocate and secure increased public funding of HIV interventions from the Ministry of Health and State Departments of Health. However, this seems to have happened at the expense of funding for vector control programs, such as those controlling mosquito vectors responsible for the transmission of dengue and Zika viruses, which mostly affected poorer communities (Lotufo 2016).

Among the poor populations, women who were either pregnant or considering pregnancy were also more vulnerable to the effects of Zika, as the virus is considered to be a teratogen (Mazzu-Nascimento et al. 2017). Government programs in Brazil that intend to provide free mosquito repellent to pregnant women do not consistently reach some of the poorest communities, such communities are remote and often lack accessible quality health care facilities due to distance and poor physical infrastructure (Hennigan 2016). Access to quality reproductive health care is essential given the accumulating evidence linking the Zika virus to a rise in cases of infants born with microcephaly—an unusually small head for age and sex (World Health Organization 2017a). Conditions related to poverty, such as poor sanitation and increased exposure to larvicides and insecticides, which may cause mutation, have also been implicated (Diniz 2016).

The relationship between the Zika virus and pregnancy is further implicated by social inequalities in the context of reproductive rights and freedoms. Access to reproductive health services, such as contraception and abortion are discouraged or illegal in many epidemic countries (Plourde and Bloch 2016). Tavares & Foster (2016) suggest that “this public health emergency offers a window of opportunity to advance national policies and practices to ensure that Brazilian women have access to a full range of reproductive health services” (p. 109) (Tavares and Foster 2016). This speaks to the inherent politicization of the Zika epidemic.

## Credibility of Evidence Informing Response Pathways

The evidence and the diversity of causal explanations of the Zika epidemic are arguably political. There are competing narratives that, if considered, justify different responses. These are exemplified in relationship to international human travel, climate change, ecosystem, and socioecology. The following is a brief overview of some theories of causation of the Zika epidemic.

International human travel is thought to have facilitated the spread of the Zika epidemic. The Zika virus was thought to have been introduced to South America between May and December 2013. During this time, there were three major international events held in Brazil—World Youth Day in July 2013, the Fifa World Cup in 2014, and the World Sprint Championship Canoe Race in Rio de Janeiro in 2014—that may have been responsible for this introduction (Possas 2016). The World Sprint Championship Canoe Race in Rio de Janeiro was attended by a team from French Polynesia, where the Zika outbreak first occurred (Possas 2016; Gautret and Simon 2016). These types of mass gatherings attracting international travelers put attendees at greater risk for transmission of communicable diseases (Gautret and Simon 2016; World Health Organization 2017b). Despite these risks, the WHO only advised pregnant women and women planning to get pregnant to avoid traveling to Zika-affected regions but did not implement a travel ban (Lima-Camara 2016).

While the climate of South America is particularly suitable for replication of the Aedes mosquito—the vector responsible for transmission of the Zika virus (Gyawali et al. 2016), climate change is thought to have resulted in extremely heavy rainfall and droughts which can support this proliferation of mosquitos, hence facilitating the spread of the Zika epidemic (Fast et al. 2015). Both the puddles created during heavy rainfall and the open barrel water storage during droughts, create ideal breeding places for mosquitos (Fast et al. 2015). Since the causes of climate change are complex, global, and political, the responses are political, often emphasizing the symptoms of climate change at a local level (in especially the poor countries), rather than addressing the causes at a global level.

A combination of an ecosystem-focused perspective and a social-anthropological lens is vital “because a pathogen requires a receptive population in order to cause disease” (Possas, 2016, p. 8) (Possas 2016). Ultimately, the need for a cross-disciplinary response pathway cannot be understated (Possas 2016).

Comprehending the diversity of causal explanations and associated responses allows for the politics of evidence to be more deeply appreciated. The production and evaluation of the evidence used to provide a causal explanation and promote a response is also political.

## Attribution of Infectious Disease Responsibility

In the case of the Zika epidemic, most of the literature on the attribution of infectious disease responsibility focused primarily on the cause, rather than the response, with most of the literature suggesting that globalization was responsible for the spread of Zika.

Although typically the blame of the origins of infectious diseases are typically placed on a cultural minority group (Hagan et al. 2015; Kobayashi et al. 2015; Phua 2015; World Health Organization 2017c), with response action emphasizing individual behavior, prevention of mosquito bites and sexual transmission (World Health Organization 2017d; Brym and Lie 2014), however, arguably, in the case of the Zika epidemic, considerable responsibility was given to the effects of globalization. Globalization has been defined in numerous ways. For example, Bryn and Lie (2014) defined it as the “rapid increases in the volume of international trade, travel, and communication [which has] broken down the isolation and independence of most countries and people” (pg. 27) (Imperato 2016). This interpretation of globalization will be used of the purposes of this review. According to the literature, the interconnectedness between countries through travel facilitated the spread of Zika beyond the Zika Forest of Uganda—where it was initially discovered in the 1940s—to South East Asia in the 1960s, the Island of Yap in 2007, French Polynesia in 2013, and South America in 2015 (Gyawali et al. 2016; Imperato 2016).

In addition to travel, other factors related to globalization, such as urbanization and human activities are also thought to have facilitated the spread of mosquito borne diseases, such as Zika. The global movement of humans and goods via international trade and the convenience of air travel have supported the ongoing spread of the Zika virus (World Health Organization 2017e). Analyses of the number of international travelers departing from Brazil and of the direction of spread of dengue and chikungunya suggest that Zika will continue to spread across South America into the Caribbean and the Southern USA (Gyawali et al. 2016).

In light of the profound influence of globalization, international human travel, climate change, and urbanization on the spread of the Zika virus, some of the literature has called for research that assesses the feasibility of a more global response to preventing spread.

## Ebola in Liberia

Table 4 summarizes the literature included in the review of Ebola in Liberia. Liberia was the focus for the purposes of this systematic review because of its high prevalence of Ebola in comparison to other affected areas. Similar to Zika, the reviewed literature spoke to all of the themes identified in the politics of epidemics literature (socioeconomic distribution of disease and the attribution of infectious disease responsibility), with the exception of decision-making in research and development.

## The Socioeconomic Distribution of Disease

Ebola virus disease has been predominantly reported in low-income countries, with the last outbreak in 2014 reported to have caused 11,323 deaths worldwide and 4809 deaths in Liberia alone (Abramowitz et al. 2015). The most affected countries typically had poor health infrastructure (Arima and Shimada 2014; Burkle and Burkle 2015; McNamara 2016; Maras and Miranda 2016). It is only during the most recent outbreak that the virus spread to high-income countries (Burkle and Burkle 2015; Stanturf et al. 2015). Within these countries, and as was exemplified in Liberia, the outbreak mostly affected those populations that were very poor, remote, and lacked proper physical infrastructure, including roads, proper sanitation, and health facilities.

The burden of the Ebola epidemic fell disproportionately on the most disadvantaged Liberians, fundamentally politicizing the epidemic. The socioeconomic distribution of Ebola was political, as defined for the purposes of this review, in that many scholars attribute its emergence to the socioeconomic conditions of Liberia. For example, poverty, and subsequent limited investment into the health system led to weak public health infrastructure, insufficient information technologies, a lack of trained personnel, inadequate case reporting, was often cited as the key vulnerability of Liberia (Burkle and Burkle 2015; McNamara 2016; Maras and Miranda 2016; Scott et al. 2016). Additional challenges included mobility of particular populations, authority distrust (Fallah et al. 2015), economic instability, and a lack of governance (McNamara 2016). These challenges intersected with other stressors, such as climate change and food insecurity to intensify the effects of the Ebola epidemic (Scott et al. 2016). Many rural communities in Liberia were particularly vulnerable, extremely poor, and lacking secure access to food and health clinics (Scott et al. 2016).

Similar to the other epidemics, people living in poverty were most vulnerable to the effects of the Ebola epidemic. Poverty facilitated the spread of the Ebola virus; thus, interventions aimed at addressing poverty would be most effective in containing further transmission (Pellecchia et al. 2015). Socioeconomic inequality was additionally evident in the “inequitable management of the dead” (Pellecchia, Crestani, Decroo, Van en Bergh, Al-Kourdi, 2015, p. 1) (Garbuglia 2016). While cremation was a less acceptable cultural practice (Kobayashi et al. 2015), it was mandated to limit further Ebola transmission (Garbuglia 2016). The new practice seemed to have impacted the economically disadvantaged who could not afford to pay for private burial services instead of cremation, aggravating socioeconomic divides (Garbuglia 2016).

These issues frame the Ebola epidemic in Liberia as political as the operational processes at the local, national, and international organizations had a negative impact on the most vulnerable Liberians. Given this context of vulnerability, many scholars investigated the response pathways undertaken.

## Credibility of Evidence Informing Response Pathways

The legitimacy and authority of the producer of evidence determines its credibility. Theoretically, the evidence used to guide response pathways during an epidemic should be credible by these measures. Most of the literature reviewed alluded to the fact that the evidence used to inform the responses during the Ebola epidemic in Liberia was inadequate, leading to a critique of global organizations’ responses. For example, some analysts argued that the epidemiological studies necessary to identify the source of Ebola virus were insufficient, leading to the ongoing emergence of the epidemic (Davies and Rushton 2016). Furthermore, the United Nation’s Security Council was criticized for not using the United Nations Mission in Liberia (UNMIL) to its full capacity in responding to the Ebola epidemic in addition to accusations of an overdue response (Roemer-Mahler and Rushton 2016).

The disorganized and delayed response has been labeled a global health governance failure by Roemer-Mahler and Rushton (2016) who argue that “the outbreak was not only a global health problem but also a global political problem” (p. 374) (Siegel 2015). Siegel (2015) echoes a similar criticism in noting that development aid was used in contexts with insufficient infrastructures for the aid to be effective and focused too heavily on issues unrelated to Ebola (Nunes 2016). The international responses are argued to be far too short term, framing the Ebola epidemic as an African, and therefore racialized problem (Jones 2011), leading to global neglect of the disease. This scholarly evaluation of global responses to the Ebola virus politicizes the epidemic, calling into question the evidence used to inform response pathways, which were largely insufficient and inappropriate for the Liberian context. More credible sources of evidence would have considered the urgency of Ebola and the unique sociopolitical context in which it was spreading.

## Attribution of Infectious Disease Responsibility

The Liberian culture was blamed not only for causing the epidemic but for interfering with control measures. Jones (2014) criticizes this “culturalist epidemiology” (pg. 1) that overlooks the wider global forces that promote the spread of Ebola, instead exoticizing Liberian culture to attribute responsibility (Blair et al. 2017). For example, traditional burial practices and the consumption of bush meat were identified as key etiological factors to the Ebola epidemic (Leach et al. 2010). Some analysts even suggest that these cultural practices, in addition to local distrust of authorities may have obstructed interventions (Phua 2015; World Health Organization 2017c; Cordner et al. 2017). Therefore, population behaviors, such as education and safe burials and cremations, were proposed as targets for intervention (Hagan et al. 2015; Alexander et al. 2015).

However, other proposed causes of the Ebola epidemic included seasonal triggers, infection of nonhuman primates, landscape modification by humans, poverty, inadequate public health infrastructure, conflict, and population growth (Farmer 1996). Failure to focus on these and narrowly focusing on cultural practices politicizes the Ebola epidemic; yet, public health authorities, governments, and academics have largely attributed disease responsibility to local culture.

## Discussion

Based on the literature on the politics of disease epidemics, four key themes emerged: (1) the socioeconomic distribution of disease, (2) decision-making in research and development, (3) the credibility of evidence that informs response pathways, and (4) the attribution of infectious disease responsibility. These were used to analyze the narratives of the politics of SARs, Zika, and Ebola in Canada, Brazil, and Liberia respectively, in the peer-reviewed literature (Table 5).

**Table 5:** The politics of epidemics: key themes by epidemic

Theme	Case-specific subtheme		
	Zika	Ebola	SARS
The social distribution of disease	-Social inequality	-Limited resourcing and vulnerability	N/A
Decision-making in research and development	N/A	N/A	N/A
Credibility of evidence informing response pathways	-Competing causal explanations and associated responses	-Global response	-Deciding how to respond
Attribution of infectious disease responsibility	-Globalization	-Culture as cause	-Capacity of the Canadian health care system

There were similarities and differences in the narratives about the different epidemics. Broadly, none of the epidemics had narratives relating to all four themes of the politics of epidemics. However, both Zika and Ebola had narratives on three of the four themes. The SARs literature addressed only two of the four themes. Notably, there was lack of relevant literature on the research and development theme.

The finding that the literature on how decisions about what research is funded and conducted during disease epidemics almost exclusively focused on the ethical implications (and did not question the potential power imbalances with regard to who identified the research issue/question and who led the research for example), was surprising, since this was a key theme in the broad politics of epidemics literature. This could, in part, be a reflection of the limitations of the search engines and strategy used in the study which excluded publications, which were deemed biomedical. Conversely, it may be a reflection of limited support for critical research and related publications.

## The Socioeconomic Distribution of Disease

A case-specific review of the literature has demonstrated the influence of power and privilege on the experience of an epidemic. In the case of Zika in Brazil, the communities most vulnerable to the virus are those with insufficient resources and infrastructure (Plourde and Bloch 2016). Consequently, Zika has been socially distributed to exacerbate conditions of poverty. However, the voices of these most affected people are drowned out by more powerful and prestigious groups. This is seen in the comparison that Lotufo (2016) makes between the politics of HIV/AIDS and the politics of Zika (Lotufo 2016). HIV/AIDS research in Brazil procured greater funding because those affected tended to be very notable Brazilians with more dominant social and politics voices (Lotufo 2016). The same pattern was reported in relationship to the Ebola outbreak in Liberia, where weak public health infrastructure aggravated Liberian experiences of Ebola (Burkle and Burkle 2015; McNamara 2016; Maras and Miranda 2016; Scott et al. 2016), while interventions targeted cultural practices, ultimately disempowering the economically disadvantaged (Garbuglia 2016).

This review found that infectious disease outbreaks disproportionately affect the poor, specifically communities with poor physical infrastructure and limited access to quality public health services. The link between income and politics of epidemics has been discussed in the social science literature, where poverty is perceived to be the greatest risk factor. For example, Farmer (1996) argued that disease outbreaks, e.g., Ebola systematically affecting poor people and are tied to regional trade networks (Alsan et al. 2011). Building on this literature, Marcella (2011) uses the term structural violence to highlight the institutional biases, inequalities, and economic policies that emanate from global centers of power and privilege, which tend to marginalize poor people during outbreaks (Leach et al. 2010). These linkages highlight social and economic inequalities (within communities, societies and countries), which are complex and often ignored by the medical (and political) communities (Alsan et al. 2011; Leach et al. 2010).

Indeed, some of the narratives criticized the (epidemiological) evidence, which tends to overlook the role of poverty in the facilitation of disease spread. For instance, focusing on pregnancy in the case of Zika in Brazil was criticized for overlooking the conditions of poverty that might also/instead be responsible for the spike in cases of microcephaly (Diniz 2016). Furthermore, responding to Ebola in Liberia with developmental aid that is not designed for contexts with insufficient infrastructure was criticized again for overlooking the role of poverty (Nunes 2016). This limited focus on the role of poverty in the peer-reviewed medical literature calls into question the politics of the research process itself. For example, what institutions are funding the research and what are the interests of the stakeholders in the research process? Who gets funding to conduct the research? What are the advantages of overlooking poverty for those producing the evidence? By disregarding the role of poverty and income inequality, epidemic responses will remain insufficient, and may, instead worsen the situation of poor populations (Mykhalovskiy and Weir 2005). Perhaps poverty remains unaddressed in epidemic responses as its origins in a neoliberalist society feel too deep to uproot.

## The Credibility of Evidence that Informs Response Pathways

Given that resources available for public health spending tend to be scarce, it is important that the risk of an epidemic is not distorted and that credible evidence on the effectiveness of the interventions is used to inform responses and justify spending. During the SARS outbreak, some of the published literature questioned the evidence on the efficacy of the proposed interventions, quarantine (Jacobs 2007) and travel advisories (Paquin 2007). In Brazil, some evidence suggests that the spike in microcephaly cases is not a consequence of the Zika virus, but rather, the result of conditions of poverty, including poor sanitation and exposure to larvicides and insecticides (Diniz 2016). This calls into question recommendations to delay pregnancy due to the Zika virus. As discussed above, the quality of response from global health organizations to the Ebola outbreak in Liberia remains debatable and questions the evidence used to inform and justify these responses.

Ironically, disease outbreaks facilitated the development of the Global Public Health Intelligence Network (GPHIN), an information sharing platform whose aim is to improve the credibility and authority of public health specialists to manage an outbreak. The platform is thought to have reduced the time between the outbreak and reporting and is thought to contribute to 40% of WHO’s early warning and played a role in SARS notification, and subsequent outbreaks (Huff and Winnebah 2015).

## Attribution of Infectious Disease Responsibility

The review clearly showed the consistency in the scholarly literature, that it is common to find that complex issues are oversimplified, whereby culture is used to justify the assigning of blame to minority groups (Leach and Tadros 2014; Lupton 2016). The review highlighted the role of politics and power in shaping different narratives, whereby powerful institutions assert particular narratives (often marginalizing the populations), which are “pushed” to frame policies, publications, interventions, and funding agendas, while the narratives of the marginalized populations (those voiced by or representing marginalized people) are marginalized (Mykhalovskiy and Weir 2005). This type of outbreak narrative was evident in the three epidemics discussed in this paper. In Toronto, SARS became a racialized disease, ultimately victimizing and excluding the Asian-Canadian community (Jacobs 2007; Ali 2008). The spread of the Zika virus in Brazil is largely attributed to the consequences of globalization, including the widening habitat of the Aedes mosquito vector and increased human and air travel (Plourde and Bloch 2016; Gyawali et al. 2016; Fast et al. 2015; World Health Organization 2017e; Ordaz-Németh et al. 2017). Cultural explanations are prominent in the outbreak narrative speaking to Ebola in Liberia, specifically the human consumption of bush meat and local/traditional burial practices that involve the touching and kissing of the deceased (Hagan et al. 2015; Kobayashi et al. 2015; Phua 2015; World Health Organization 2017c; Washington and Meltzer 2015; Ng 2008).

When responsibility for the origin of the epidemic is reduced to cultural and ethnic minority groups, for example, the Asian community during the SARS epidemic, South American women living in poverty during the Zika epidemic, and communities engaging in traditional Liberian cultural practices during the Ebola epidemic, this further marginalizes the already vulnerable populations. It is important that emphasis is placed on the extreme vulnerability of these groups to an infectious disease outbreak, rather than placing blame and ultimately exacerbating experiences of oppression.

## Limitations

The findings in this paper should be interpreted with caution. First, this systematic review relied upon published peer-reviewed literature. This overlooked documents, such as WHO reports, government documents, and books, which might have contained relevant information. For example, we are aware of critical texts in the form of books, which may have enriched this manuscript—specifically, Ng (2008) and Teo, Yeoh, and Ong (2008) provide insight into how other cities affected by SARs attributed responsibility, talked about attribution of risk and responsibility for the disease; (Teo et al. 2008; Keil and Ali 2008) Teo, Yeoh, and Ong (2008) point to Singapore’s attribution of responsibility and credibility of evidence; (Keil and Ali 2008), while Keil and Ali (2008) also note the racialization of the SARS epidemic in Toronto, reflecting on the stigmatization of Toronto’s Chinese and South Asian communities (King 2008). However, King’s (2008) work positions globalization as both responsible for causing and responding to infectious disease outbreaks (Ali et al. 2016), such narratives are thought to create space to better understand how such processes might be repurposed as public health solutions. Finally, Ali, Dumbuya, Hynie, Idahosa, Keil, and Perkins (2016) ground the Ebola outbreak in Liberia in the context of colonial legacies, specifically emphasizing that global public health responses were political in that the establishment of the public health infrastructure tasked with responding to Ebola was influenced by social inequality, colonialism, and racism (). By adopting a social science perspective, Ali et al. (2016) unpack the diverse factors—social, political, environmental, medical, and legal—that facilitated the escalation of the Ebola crisis (). However, since the scope of this paper was limited to peer-reviewed journal publications and one outbreak from each income context as an illustration, such information (from books and from other contexts) although relevant, was beyond the paper’s scope.

Another limitation is the time frame of the study. Scholarly literature is consistently evolving, and in the specific case of the Zika epidemic, which was considered a public health emergency of international concern at the time of data collection, new and relevant research was being produced after data collection ended. For feasibility purposes, data collection ended in June 2017.

It is also important to note that research and research publications sometimes tends to be biased and may marginalize the narratives by or representing the most vulnerable poor populations and political topics. Furthermore, social science literature that did not fit the definition of politics as articulated for the purposes of this review was excluded. A future review might seek to unpack the themes that emerge from this additional literature.

## Conclusion

This systematic review of the politics of three different outbreaks in three different social economic contexts revealed that the politics of epidemics are—to an extent—universal. However, the manner by which the politics are played out vary by the income setting, the political themes that speak to general epidemics were found to be uniquely enacted during the SARS outbreak in Toronto, the Zika outbreak in Brazil, and the Ebola outbreak in Liberia.

Perhaps the most universal finding of this systematic review is the role of social and economic inequality, including poverty during an epidemic. Regardless of the national income setting, minority and marginalized communities are the most devastated by an epidemic. If organizations and governments are to adequately respond to these individuals and communities, it is critical that narratives of those most vulnerable to an epidemic—specifically poor communities—are represented in the mainstream media as well as in the peer reviewed published literature—especially, the epidemiological and medical literature that tends to influence health programming and policy-making.
